# A conformational rearrangement of the SARS-CoV-2 host protein sigma-1 is required for antiviral activity: insights from a combined in-silico/in-vitro approach

**DOI:** 10.1038/s41598-023-39662-w

**Published:** 2023-08-07

**Authors:** Francesca Serena Abatematteo, Pietro Delre, Ivan Mercurio, Veronica V. Rezelj, Dritan Siliqi, Stephanie Beaucourt, Gianluca Lattanzi, Nicola Antonio Colabufo, Marcello Leopoldo, Michele Saviano, Marco Vignuzzi, Giuseppe Felice Mangiatordi, Carmen Abate

**Affiliations:** 1 Dipartimento di Farmacia-Scienze del Farmaco, Università degli Studi di Bari Aldo Moro, Via Orabona, 4, 70125 Bari, Italy; 2 Consiglio Nazionale delle Ricerche (CNR), Istituto di Cristallografia, Via Amendola 122/O, 70126 Bari, Italy; 3 Department of Environmental, Biological and Pharmaceutical Sciences and Technologies, University of Campania “Luigi Vanvitelli”, Via Antonio Vivaldi 43, 81100 Caserta, Italy; 4 Viral Populations and Pathogenesis Unit, UMR 3569, CNRS, Institut Pasteur, Paris, France; 5 Department of Physics, University of Trento, Via Sommarive 9, 38123 Povo-Trento, Italy; 6 TIFPA Trento Institute for Fundamental Physics and Applications, Via Sommarive 9, 38123 Povo-Trento, Italy; 7 Consiglio Nazionale delle Ricerche (CNR), Istituto di Cristallografia, Via Vivaldi 43, 81100 Caserta, Italy; 8 A*STAR Infectious Diseases Labs (A*STAR ID Labs), Agency for Science, Technology and Research (A*STAR), 8A Biomedical Grove, Immunos #05-13, Singapore, 138648 Singapore

**Keywords:** Computational biology and bioinformatics, Drug discovery, Medicinal chemistry

## Abstract

The development of effective drugs to treat coronavirus infections remains a significant challenge for the scientific community. Recent evidence reports on the sigma-1 receptor (S1R) as a key druggable host protein in the SARS-CoV-1 and SARS-CoV-2 interactomes and shows a potent antiviral activity against SARS-CoV-2 for the S1R antagonist PB28. To improve PB28 activity, we designed and tested a series of its analogues and identified a compound that is fourfold more potent against SARS-CoV-2 than PB28 itself. Interestingly, we found no direct correlation between S1R affinity and SARS-CoV-2 antiviral activity. Building on this, we employed comparative induced fit docking and molecular dynamics simulations to gain insights into the possible mechanism that occurs when specific ligand–protein interactions take place and that may be responsible for the observed antiviral activity. Our findings offer a possible explanation for the experimental observations, provide insights into the S1R conformational changes upon ligand binding and lay the foundation for the rational design of new S1R ligands with potent antiviral activity against SARS-CoV-2 and likely other viruses.

## Introduction

Despite the increasingly likely prospect of ending the coronavirus disease 2019 (COVID-19) pandemic with the aid of approved vaccines, we are still experiencing waxing and waning of pandemic patterns, with several viral variants that have followed and will likely continue to emerge. The long-term effects of COVID-19, known as "long COVID," are still under evaluation and include detrimental effects on the central nervous system (CNS), resulting in neurological and psychiatric symptoms. SARS-CoV-2 is the 7th coronavirus to have established respiratory infection between humans. Therefore, developing effective therapies against coronavirus infections remains a critical challenge for the scientific community. In the last two decades, three zoonotic spill overs of coronaviruses to humans have caused significant epidemics. The severe acute respiratory syndrome (SARS) outbreak of 2003 resulted in over 8,000 human infections and approximately 800 deaths^1^, the Middle East respiratory syndrome (MERS) outbreak of 2012 saw around 2,500 confirmed cases and 858 deaths^2^. Currently, the ongoing coronavirus disease 2019 (COVID-19) pandemic has resulted in over 760 million confirmed cases and more than 6.8 million deaths^3^. It is reasonable to postulate that the current pandemic caused by a coronavirus is not the first, nor will it be the last. For this reason, a global effort has been taken to prevent the world from being caught unprepared again. Several in-silico studies^4–7^ as well as clinical trials^8^ have been conducted to find effective drugs against COVID-19 through repurposing. The RNA-dependent viral polymerase inhibitor Remdesivir, immunomodulators such as monoclonal antibodies (e.g., Tocilizumab), and kinase inhibitors like Baricitinib have been approved for treating infected patients who are at high risk of progressing to severe COVID-19. Other monoclonal antibodies targeting the spike protein, as well as antiviral drugs like Paxlovid and Molnupiravir, have also received emergency use authorization from the Food and Drug Administration (FDA)^9,10^. However, the use of monoclonal antibodies is limited by the high frequency of non-susceptible viral variants. Therefore, the FDA has restricted the use of certain monoclonal antibodies in geographic regions characterized by a low frequency of such non-susceptible variants^11^. The approved antiviral drugs were discovered through drug repurposing approaches that aimed to inhibit viral proteins and counteract replication. However, targeting viral components such as viral enzymes with a drug may lead to the development of resistance due to the high rate of viral mutations^12,13^. As a result, an ideal broad-spectrum coronavirus antiviral should be able to target host components crucial to the virus's replication cycle or the development of the disease in the host, reducing the likelihood of drug resistance. Based on this evidence, a study published in Nature^14^ identified the SARS-CoV-2 interactome with 66 druggable host targets, including the endoplasmic sigma receptors. Through affinity-purification mass spectrometry, it was found that the SARS-CoV-2 protein NSP6 interacts with the sigma-1 (S1R) receptor, while ORF9c interacts with the sigma-2 (S2R) receptor. The researchers then tested several drugs already in clinical use for other conditions and known to act on the identified druggable host proteins. Among the tested molecules, **PB28**, a S1R antagonist and S2R agonist, showed most potent anti-SARS-CoV-2 activity in vitro, with an IC_90_ value of 0.28 μM. Subsequent studies, using genomic and proteomic approaches, identified S1R as a crucial host dependency factor. Specifically, studies showed that knocking out (KO) or knocking down (KD) S1R prevented the infection of cells (such as Caco-2 and A549-ACE2) by SARS-CoV-2. Conversely, KO and KD of S2R did not have the same effect^15^. Importantly, S1R displayed the same effect in both SARS-CoV-1 and SARS-CoV-2 infected cells, preventing the infection when knocked out. A retrospective study analysing the outcomes of approximately 800,000 infected patients undergoing typical or atypical antipsychotic treatment provided hints of the in vivo effectiveness of S1R modulation as anti-SARS-CoV-2 therapy^16^. It is worth noting that typical antipsychotics have an affinity for the S1R, whereas atypical ones do not. The retrospective study showed that a high percentage of infected patients undergoing treatment with typical antipsychotics had better outcomes. Several clinical studies, both retrospective and prospective, have been conducted with fluvoxamine, a serotonin reuptake inhibitor and potent S1R agonist. These studies suggest that fluvoxamine may act as a prophylactic drug for early-stage SARS-CoV-2-infected patients^17,18^. Furthermore, a S1R polymorphism (rs17775810) has been linked to a reduced death rate associated with COVID-19, with the TT genotype performing better than the CT and CC genotypes, thus providing additional evidence for the S1R involvement in SARS-CoV-2 replication^19^. It is noteworthy that S1R ligands have been found to exhibit antiviral activity against other viruses as well^20^. The various findings about the involvement of S1R in coronavirus infections and the positive results obtained from PB28 and other S1R ligands already in clinical use, have sparked interest in further exploring the role of S1R in these infections. This could hopefully lead to the development of novel ligands with the potential to provide therapeutic options against mutant strains that may not respond to existing vaccines and therapies. To develop novel and more potent anti-coronavirus agents, we tested a chemical library of **PB28** analogs, focusing on those with high S1R affinity and we identified one agent that is 4-fold more potent against SARS-CoV-2 than **PB28**. However, we did not observe a direct correlation between S1R affinity and SARS-CoV-2 antiviral activity, suggesting the presence of a mechanism that may only occur if specific ligand–protein interactions take place. To investigate these interactions, we used molecular modelling and analysed the obtained data that will be useful to design new and more potent anti-SARS-CoV-2 S1R ligands^21^.

## Materials and methods

### Synthesis

All SR ligands investigated were produced according to the literature reported for each compound in Tables 1, 2, 3, 4. They were obtained with a purity > 95%.

**Table 1:** S1R/S2R affinities and anti-SARS-CoV-2 activity of a first set of **PB28** analogs.

General structure of PB28 and its analogs						

Compound	A or B	R	n	^A^*K*_i_ nM S1R	^B^*K*_i_ nM S2R	^C^IC_50_ (μM) SARS-CoV-2
**PB28**^39^	A	5-OCH_3_	3	0.38	0.68	0.299
(-)-*R*-**PB28**^39^	A	5-OCH_3_	3	0.63	0.49	0.309
( +)-*S*-**PB28**^39^	A	5-OCH_3_	3	0.13	1.18	0.455
**1**^40^	A	5-OCH_3_	0	0.40	7.90	NOT ACTIVE
**2**^40^	A	5-OCH_3_	1	0.31	16.4	5.90
**3**^40^	A	5-OCH_3_	2	1.57	21.1	12.57
**4**^40^	A	5-OCH_3_	4	1.54	3.58	0.320
**5**^40^	A	5-OCH_3_	5	1.52	0.35	0.19
**6**	A	5-OCH_3_	6	3.07	103	1.32
**7**^40^	A	H	3	0.61	0.68	0.438
**8**^40^	A	H	4	0.036	14.6	0.317
**9**^40^	A	H	5	1.45	7.85	0.19
**10**^40^	A	6-OCH_3_	3	0.36	5.42	0.47
**11**^40^	B	H	3	2.16	0.69	0.94
**12**^40^	B	H	5	2.40	0.57	0.52
**13**^40^	B	5-OCH_3_	3	1.57	9.24	0.306
**14**^41^	B	6-OCH_3_	3	3.16	9.02	NOT ACTIVE
**15**^41^	A	5-OH	3	5.40	2.66	NOT ACTIVE
**16**^41^	A	6-OH	3	0.69	1.12	NOT ACTIVE
**17**^41^	B	6-OH	3	6.78	26.4	NOT ACTIVE
**18**^41^	B	7-OH	3	5.48	11.8	NOT ACTIVE

^A,B^*K*i values represent the mean of three experiments in duplicate, ± SEM is reported in the cited manuscript; ^A^*K*i values from radioligand binding assay: [^3^H]-( +)-Pentazocine on guinea pig brain according to the generally accepted protocol; ^B^*K*i values from radioligand binding assay: [^3^H]-DTG on rat liver masking with (+)-Pentazocine according to the generally accepted protocol; ^C^Detection of viral genomes by RT-qPCR on Vero E6 cells infected with SARS-CoV-2 virus^14^. Listed values are one experiment that is representative of experiments performed at least three times.

**Table 2:** SR ligands with chemotype different from **PB28**.

Compound		^A^*K*_i_ nM S1R	^B^*K*_i_ nM S2R	^C^IC_50_ (μM) SARS-CoV-2
**19**^42^		2435	4.24	NOT ACTIVE
**20**^42^		311	3.56	NOT ACTIVE
**21**^42^		709	4.84	NOT ACTIVE
**22**^42^		3987	1.42	NOT ACTIVE

^A^^,B^*K*i values represent the mean of three experiments in duplicate, ± SEM is reported in the cited manuscript; ^A^*K*i values from radioligand binding assay: [^3^H]-( +)-Pentazocine on guinea pig brain according to the generally accepted protocol; ^B^*K*i values from radioligand binding assay: [^3^H]-DTG on rat liver masking with ( +)-Pentazocine according to the generally accepted protocol; ^C^Detection of viral genomes by RT-qPCR on Vero E6 cells infected with SARS-CoV-2 virus^14^. Listed values are one experiment that is representative of experiments performed at least three times.

**Table 3:** S1R/S2R affinities and anti-SARS-CoV-2 activity of a second set of **PB28** analogs.

**A.** *Replacement of the cyclohexyl ring*					
General structure					

Compound	A	^A^*K*_i_ nM S1R	^B^*K*_i_ nM S2R	^C^IC_50_ (μM) SARS-CoV-2	
**23**^43^	CH_3_-	17.8	663	5.78	
**24**^43^	CH_3_CH_2_CH_3_-	9.6	42.5	2.26	
**25**^43^	(CH_3_CH_2_)_2_CH-	12.0	12.7	NOT ACTIVE	

**B.** *Insertion of Heteroatoms*					
General structure					

	A	X			
**26**^44^	CH_2_	N	23.9	8.49	0.30
**( +)-R-26**^44^	CH_2_	N	13.6	14.4	0.08
**(-)-S-26**^44^	CH_2_	N	23.3	8.52	0.41
**27**^44^	CH_2_	NCH_3_	4.17	15.3	1.27
**28**^44^	CH_2_	O	3.90	9.03	5.83
**29**^44^	O	CH_2_	12.5	9.72	10.49
**30**^44^			5.44	18.5	2.1
**31**^44^			65%**	2.40	NOT ACTIVE

**C.** *Replacement of the tetralin core*					
General structure					

**32**^45^			3450	12.6	1.18
**33**^46^			0.25	4.70	NOT ACTIVE
**34**^47^			24.7	26.6	NOT ACTIVE
**35**^48^			12.0	16.0	NOT ACTIVE
**36***cis*^49^			0.042	3.66	NOT ACTIVE
**36***trans*^49^			0.045	4.77	NOT ACTIVE
**37**c*is*^49^			0.81	1.59	2.349
**37***trans*^49^			0.23	1.21	2.313
**38***cis*^49^			0.26	7.92	NA
**38***trans*^49^			0.32	0.21	NA

^A,B^*K*i values represent the mean of three experiments in duplicate, ± SEM is reported in the cited manuscript; ^A^*K*i values from radioligand binding assay: [^3^H]-( +)-Pentazocine on guinea pig brain according to the generally accepted protocol;^B^*K*i values from radioligand binding assay: [^3^H]-DTG on rat liver masking with ( +)-Pentazocine according to the generally accepted protocol; ^C^Detection of viral genomes by RT-qPCR on Vero E6 cells infected with SARS-CoV-2 virus^14^. Listed values are one experiment that is representative of experiments performed at least three times.

**Table 4:** Reference subnanomolar S1R ligands.

		^A^*K*_i_ Nm S1R	^B^*K*_i_ nM S2R	^C^IC_50_ (μM) SARS-CoV-2
**PB190**^50^		0.42	36.3	NOT ACTIVE
**PB212**^50^		0.030	17.9	NOT ACTIVE
**39**^50^		0.35	238	NOT ACTIVE

^A^^,B^*K*i values represent the mean of three experiments in duplicate, ± SEM is reported in the cited manuscript; ^A^*K*i values from radioligand binding assay: [^3^H]-( +)-Pentazocine on guinea pig brain according to the generally accepted protocol; ^B^*K*i values from radioligand binding assay: [^3^H]-DTG on rat liver masking with ( +)-Pentazocine according to the generally accepted protocol; ^C^Detection of viral genomes by RT-qPCR on Vero E6 cells infected with SARS-CoV-2 virus^14^. Listed values are one experiment that is representative of experiments performed at least three times.

### Biology

#### Materials for antiviral activity assays

VeroE6 (ATCC #CRL-1586) and A549-ACE2, a human lung epithelial cell line provided by the lab of Olivier Schwartz that is overexpressing the SARS-CoV-2 receptor, were cultured in DMEM (Gibco #31,966,021) supplemented with 10% FBS (Gibco #A3160801) and penicillin/streptomycin (100 U/mL and 100 µg/mL, Gibco #15,140,122) at 37 °C in a 5% CO2 atmosphere. In order to maintain the selection of the ACE2 clones, Blasticidin (10 µg/mL – Sigma Aldrich #SBR00022-10ML) was added to the media.

SARS-CoV-2 BA.1 strain (GISAID ID: EPI_ISL_6794907) was kindly provided by the Virus and Immunity Unit (Institut Pasteur, PMID: 35,322,239). Viral stocks were generated by infecting VeroE6 cells at a multiplicity of infection (MOI) of 0.01 in DMEM supplemented with 2% of FBS and 1 µg/mL of TPCK-Tryspin (Sigma-Aldrich #1426-100MG). Supernatant was harvested 3 days post-infection (p.i.) and stored at − 80 °C.

#### Antiviral activity assays

A549-ACE2 cells were seeded in 96 well plates at a concentration of 1.5E4 cells per well in DMEM supplemented with 10% of FBS and incubated overnight at 37 °C, 5% CO_2_.

Two hours prior to infection, the supernatant was replaced with 100 uL of DMEM – 2% FBS containing the compound of interest at a range of concentrations (100 µM, 50 µM, 20 µM, 10 µM, 1 µM or 0,1 µM); or an equivalent volume of DMSO (Sigma Aldrich—#D2650), vehicle used as a control. At the time of infection, the media was replaced with virus inoculum (MOI = 0.1 PFU/cell).

Following a one-hour adsorption at 37 °C, the virus inoculum was replaced by 200 µL of drug- (or vehicle-) containing media and cells were incubated for an additional 72 h at 37 °C, 5% CO_2_. The supernatants were then collected for RT-qPCR and cell viability assay.

#### Virus quantification by RT-qPCR

72 hpi, supernatants were collected and inactivated 10 min at 95 °C to further be used for RT-qPCR. SARS-CoV-2 specific primers targeting the N gene region: 5’TAATCAGACAAGGAACTGATTA-3’ (Forward) and 5’CGAAGGTGTGACTTCCATG-3’ (Reverse) were used with the Luna Universal One-Step RT-qPCR kit (New England Biolabs, #E3005) in an Applied Biosystems QuantStudio 6 thermocycler with the following cycling conditions: 55 °C for 10 min, 95 °C for 1 min, and 40 cycles of 95 °C for 10 s followed by 60 °C for 1 min. The number of viral genomes is expressed as PFU equivalents/mL and calculated by performing a standard curve with RNA derived from a viral stock with a known viral titer. Datas was fit using nonlinear regression and IC_50_s for each experiment were determined using GraphPad Prism version 8.1.0 (San Diego, CA).

#### Cell toxicity assay

Cell viability was assessed in drug treated cells by using Cell TiterGlo following the manufacturer’s instructions (Promega #G7570). Luminescence was measured in a Tecan Infinity 2000 plate reader.

Percentage of viability was calculated relative to untreated cells and cells lysed with 20% of ethanol.

### Computational details

#### Docking studies

**PB28**, **10**, **14** and **16** were docked on the recently published X-ray structure of S1R in complex with haloperidol (**co-x**) (resolution 3.08 Å – pdb code: 6DJZ)^22^. The retrieved .pdb file was prepared using the Protein Preparation Wizard tool, available from the Schrodinger Suite 2021–2^23^ for adding missing hydrogen atoms, reconstructing incomplete side chains, assigning favourable protonation states at physiological pH and performing a force field based minimization of the 3D protein structures. All ligands were prepared using the LigPrep tool for generating all the possible ionization states and tautomers at a pH value of 7.0 ± 2.0^24^. The obtained files were employed for docking simulations performed by Grid-based ligand docking with energetics^25^. In particular, we performed Induced Fit Docking (IFD)^26^ simulations in order to properly take into account putative conformational rearrangements of the protein binding site during molecular recognition. Docking simulations were performed using the SP mode and all the default settings, building a cubic grid centered on the co-x and having an inner box of 10 Å × 10 Å × 10 Å and an outer box of 30 Å × 30 Å × 30 Å. In particular, these steps were followed: i) docking of each ligand using a softened potential and retaining only those poses (maximum 20) that meet specific criteria based on Coulomb-vdW (less than 100) and H-bond (less than –0.05) thresholds; ii) prediction of the best orientation of that residues within a certain distance (5 Å) of any ligand pose; iii) minimization of the obtained complexes to accommodate the ligand structure in the induced-fit protein conformation; iv) redocking using the standard precision (SP) protocol of each protein–ligand complex structure within a specified energy of the lowest-energy structure (30 kcal/mol), v) computation of the binding energy (IFDScore) for each output pose. The top-scored docking complexes were subjected to molecular mechanics/generalized Born surface area calculations (MM-GBSA) using the OPLS2005 force field^27^. Notice that during this calculation, no flexibility was allowed for the residues of the binding site since a conformational rearrangement of the binding site had already been considered during the IFD simulations.

#### Model system preparation

The **S1R-(*S*)-PB28** and **S1R-(*R*)-16** complexes returned by IFD simulations were subjected to Molecular Dynamics (MD) simulations to get insights into putative effects of the ligand binding on S1R conformation. Using the system builder^28^, the complexes were inserted into a pre-equilibrated (T = 310 K) 1-palmitolyl-2-oleoyl-sn-glycero-3-phosphoethanolamine (POPE) lipid bilayer and fully solvated into a minimized, orthorhombic TIP3P water-box (10 × 10 × 10 Å^3^). Na^+^ and Cl^−^ ions were added generating a 150 mM ionic concentration. In doing that, we obtained two electrically neutral systems including ~ 90,000 atoms. The default OPLS4 force field^29^ was used for both protein and ligands.

#### MD simulation protocol

Simulations were performed on GPUs by using Desmond 4.2, implemented in the Schrodinger Suite 2022–4^28^, as software program. A non-bonded cut-off of 9 Å was used. All the prepared systems were minimized, equilibrated and simulated using an isothermalisobaric ensemble (NPγT, P = 1 atm, T = 300 K) with a Nosè–Hoover thermostat^30^ and a Martyna-Tobias-Klein barostat^31^. We performed a 200 ns-long MD simulation for each investigated complex using a time step equal to 2 fs and storing the coordinates with a recording interval of 100 ps. In doing that, 2001 frames were generated and analysed for each system.

#### MD simulation analysis

The obtained trajectories were analysed using the trajectory player available in the Schrodinger Suite 2022–4^28^. More specifically, the Root Mean Square Deviation (RMSD) and Root Mean Square Fluctuations (RMSF) of the alpha-carbon atoms of the protein were computed using the 'Compute Properties Over Trajectory' tool. All the results were saved in a .csv file used as input file for an in-house R script written to generate the corresponding 2D plots.

#### Molecular properties calculation

We utilized QikProp^32^ and Epik 7^33^ to predict the octanol/water partition coefficient (referred to as logP) and the macro-pKa of all investigated compounds in this study. Specifically, Epik 7 employs a reliable machine-learning model to predict the pK_a_ for all the reasonable ionization sites within each molecule^33^.

## Results and discussion

### *PB28 derivatives and anti-SARS-CoV-2 *in vitro* activity*

To investigate a range of biological features that affect anti-SARS-CoV-2 activity, we constructed a library of SR binders consisting of several S1R and S2R ligands closely related to **PB28**. This library included ligands with an affinity for SR subtypes ranging from subnanomolar to micromolar *K*_i_ values, with varying degrees of S1R *vs* S2R selectivity. We also investigated S1R or S2R ligands with different chemotypes than **PB28** to determine whether the anti-SARS-CoV-2 activity was specifically associated with a particular structure type. While the literature has extensively discussed the structure-affinity relationship studies of the S1R and S2R ligands reported in Tables 1, 2, 3, 4, as recently reviewed^34^, in this study we present their activity as IC_50_ values against SARS-CoV-2 virus in Vero E6 cells and correlate the obtained values with the known affinities of SRs. As **PB28** is a chiral compound, we investigated the corresponding pure enantiomers, namely (-)-(*R*)-**PB28** and ( +)-(*S*)-**PB28**. Notably, we did not observe a substantial change in antiviral activity compared to the racemate, which is in line with the measured S1R and S2R affinities. It is worth noting that the activity of the compounds is basically unaffected when the alkyl chain is elongated from propyl to hexyl, with hexyl being only threefold less potent (**6**). However, lower homologs exhibit reduced activity, with a 20- or 40-fold reduction observed for compounds **2** and **3**, respectively, which feature a methylene or ethylene linker, respectively. Furthermore, despite the subnanomolar affinity at the S1R returned by compound **1**, the absence of a linker between the tetralin nucleus and the piperazine moiety results in a loss of activity. The change in position of the methoxy group (6–OCH_3_, **10**), on the other hand, did not affect the activity. Additionally, the replacement of the tetralin ring with a naphthalene nucleus, along with the presence (5–OCH_3_, **13**) or absence (**11**, **12**) of the methoxy group, did not alter the activity. A notable finding was that changing the position of the methoxy group in the naphthalene series resulted in a complete loss of activity, despite the compound's 1-digit nanomolar affinity for the S1R (for example, compound **14** with a methoxy group at position 6). Interestingly, replacing the methoxy group with a hydroxyl group in either the 5- or 6-position also led to a complete loss of antiviral activity, despite high S1R affinity observed for both the tetralin (**16**, **15**) and naphthalene (**17**, **18**) series. The above compounds generally exhibit subnanomolar or 1-digit nanomolar affinity at the S1R and slightly lower affinity at the S2R, except for the hexyl-bearing compound **6**. We also tested a small series of S2R ligands that have no or negligible affinity at the S1R (Table 2). These compounds feature the 6,7-dimethoxytetrahydroisoquinoline as the basic moiety instead of the cyclohexylpiperazine, and a dihydroisoquinolinone (**19**, **20**, **21**) or tetrahydroquinoline (**22**) as the hydrophobic moiety. They were all inactive in the anti-viral assay, which supports the conclusion that S2R is not involved in the anti-SARS-CoV-2 activity. However, based on the collected data, it is not possible to establish a correlation between the anti-viral activity and the S1R affinity of these compounds, as ligands with high affinity for S1R may result in highly active or inactive compounds. Confounding factors in the anti-SARS-CoV-2 assays have been reported due to phospholipidosis^35^ induced by cationic amphiphilic drugs, such as some SR ligands. However, given the structural similarity of the compounds analysed, it would be expected that all of them have the potential to induce phospholipidosis and show antiviral activity in vitro, since this phenomenon is a common side effect of many cationic amphiphilic drugs and is mainly related to their lipophilicity^36^. To investigate this further, we have expanded our analysis to include other derivatives of **PB28** with varying lipophilicities and chemical structures different from **PB28** (Tables 3 and 4). Replacing the cyclohexyl ring with shorter alkyl chains, such as methyl (**23**) or *n*-propyl (**24**), results in a 20- or 8-fold reduction in anti-SARS-CoV-2 activity, respectively (Table 3A). However, the compound (**25**) with a 3-pentyl group in place of the cyclohexyl one shows no anti-SARS-CoV-2 activity, despite its better match with the **PB28** physicochemical properties (Table 3A). Compounds with heteroatoms in the linker or a tetralin portion with lower lipophilicity than **PB28** were also evaluated (Table 3B and Table 3C). Various modifications were made to the structure of the compounds, and as a result, their anti-SARS-CoV-2 activity was affected differently. Among less lipophilic compounds, those with N-heteroatoms displayed activity comparable to **PB28**, with the (*R*)-enantiomer exhibiting a fourfold increased activity (**26**), while methylation of the NH group (**27**) or aromatization of the tetralin core to naphthalene (**30**) led to a 4- to 7- fold reduction in activity (Table 3B). However, the insertion of an oxygen atom in the linker or the tetralin core caused a drop-in activity, with a significant reduction for the tetralin-bearing compounds (**28** and **29**) and a complete loss for the naphthalene counterpart (**31**) (Table 3B). Furthermore, replacement of the tetralin nucleus with other structures, such as carbazole, cyclohexane, dihydro-isoquinolinone, pyrido-ozaxinone, or differently substituted benzene-cyclohexyl ring, led to inactive compounds in the anti-SARS-CoV-2 assay, except for **37***cis* and **37***trans* couple, whose IC_50_ value is in the 2 µM range (Table 3C). Again, all these compounds display affinity at the SRs spanning from subnanomolar to micromolar. It is interesting to note that **PB190** and **PB212**, which are known to have a subnanomolar affinity as a S1R agonist and antagonist, respectively, did not demonstrate any antiviral effect in the performed assay, nor did their congener **39** (Table 4). LogP and pKa values were calculated for all the compounds (Tables 1, 2, 3, 4) and reported for comparison (Table S1). The former values ranged from 3.30 to 5.51 while the latter from 4.03 to 13.31. Overall, the data obtained suggest that: *(i)* S1R ligands with *K*_i_ values ranging from subnanomolar to two-digit nanomolar have anti-SARS-CoV-2 activity, *(ii)* pure S2R ligands do not exhibit antiviral activity, and *(iii)* some high-affinity S1R ligands may not possess anti-SARS-CoV-2 activity. Notably, hydroxy-derivatives of **PB28** (**15**, **16**, **17** and **18**, Table 1), despite having low nanomolar S1R affinity, are inactive in the anti-SARS-CoV-2 assay. Additionally, the results obtained from **PB190** and **PB212** suggest that the measured antiviral activities cannot be justified solely by the affinity or functional activity, when available, of S1R ligands. S1R has been defined as a pluripotent modulator that mainly resides at the interface between mitochondria and endoplasmic reticulum. It is able to translocate and interact with several proteins modulating their functions thus resulting in multiple functions and therapeutic applications^37^. It should be noted that the currently available functional assays may not account for all possible S1R protein interactions, making it difficult to assign the terms "agonist" or "antagonist" to S1R ligands^38^. Based on the obtained data and reported conformational changes described in the literature, we have postulated that the S1R ligands may induce a conformational rearrangement of the S1R upon binding to produce the antiviral effect, possibly by interacting with NSP6.

### Computational studies

#### Induced fit docking (IFD) simulations

**Figure 1:**
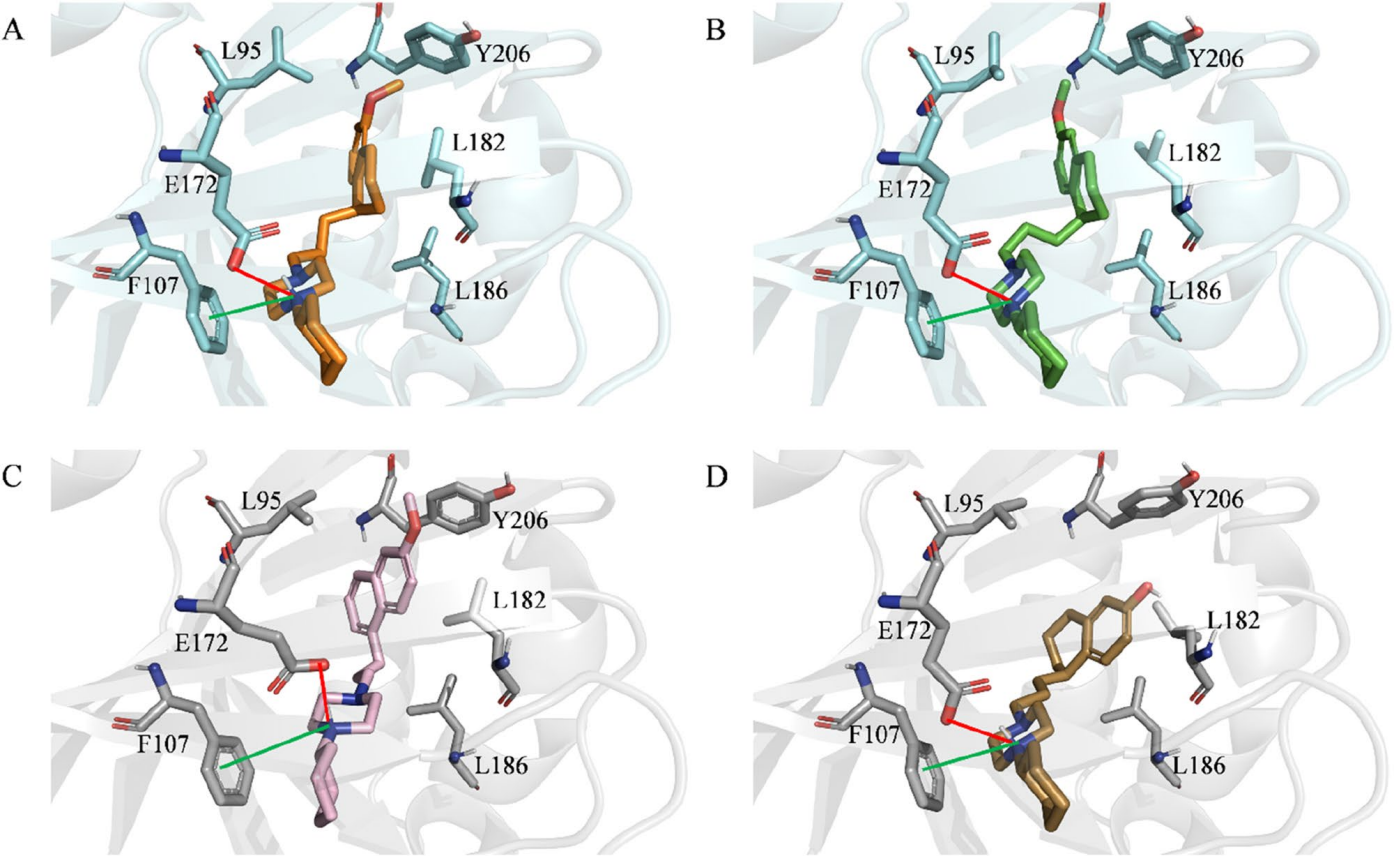
Top-scored docking poses within the binding pocket of S1R of: (**A**) (*S*)-**PB28**; (**B**) (*S*)-**10**, (**C**) **14**, (**D**) (*R*)-**16**. For the sake of clarity, only polar hydrogen atoms are shown. Important residues are rendered as sticks while the proteins are represented as cartoons. Salt-bridge and cation-pi interactions are depicted by a red and green line, respectively.

**Figure 2:**
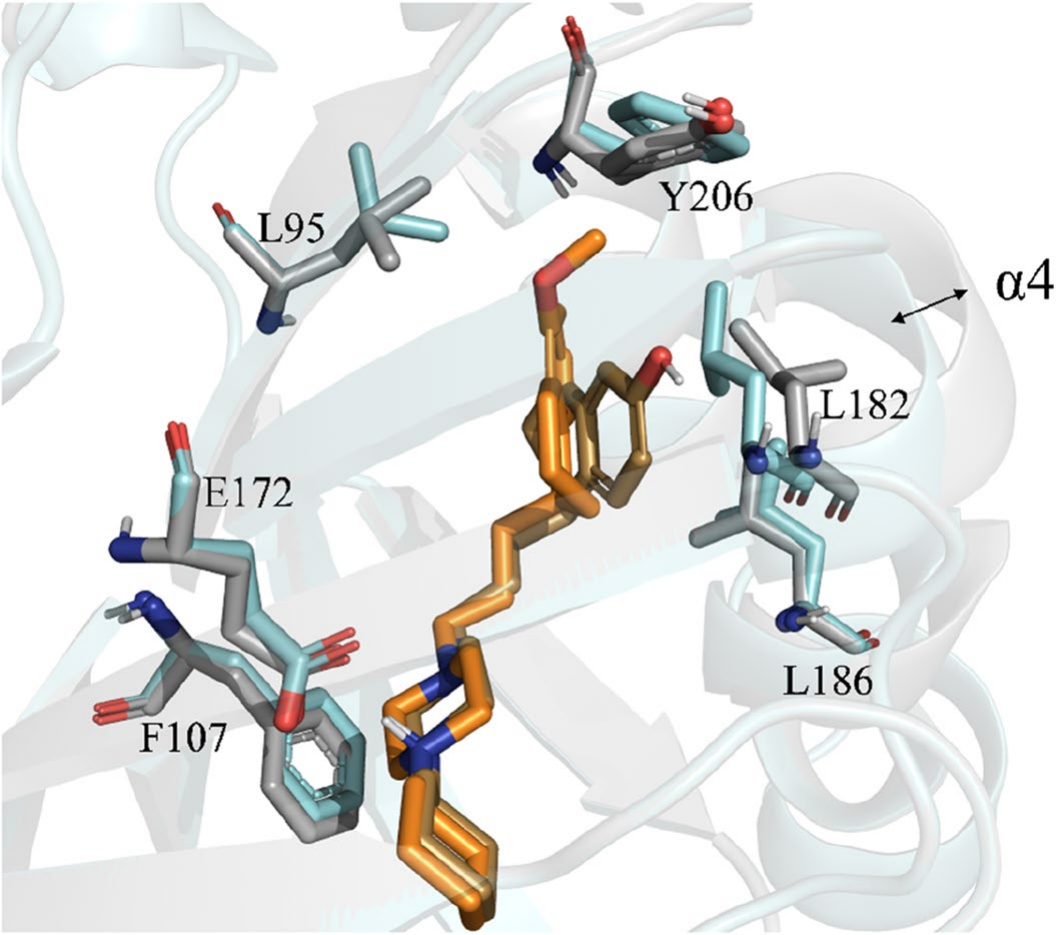
Superposition of the **S1R **(cyan)-(***S*)-PB28 **(orange) and **S1R **(gray)-(***R*)-16** (sand) complexes. The observed α4-helix shift is indicated by a bidirectional black arrow. For the sake of clarity, only polar hydrogen atoms are shown. Important residues are rendered as sticks while the proteins are represented as cartoons.

Based on the experimental data obtained, which suggests the presence of a conformational change responsible for the observed antiviral activities, we conducted IFD simulations to gain new molecular insights into potential conformational crosstalks between S1R and the ligands in our series. Additionally, we focused our attention on three ligands from our series that were selected as molecular probes due to their high structural similarity with the reference compound **PB28** (*K*_i_ (S1R) = 0.69 nM) and strong affinity for S1R. Specifically, we investigated the effect of: *(i)* a methoxy shift from the 5- to 6-position (**10**, *K*_i_ (S1R) = 0.38 nM); *(ii)* the substitution of the tetralin core with a naphthalene one (**14**, *K*_i_ (S1R) = 3.16 nM); and (*iii)* the replacement of the methoxy substituent in position 6 with a hydroxy group (**16**, *K*_i_ (S1R) = 0.69 nM). Although these compounds share a high structural similarity, only **PB28** and **10** were found to be responsible for antiviral activities (Table 1). Figure 1 displays the top-scored docking poses returned by the IFD protocol. As expected, the predicted binding mode for each investigated compound closely resembles that observed experimentally for the co-crystallized ligand, based on the crystal structure. Furthermore, all investigated ligands produced high MM-GBSA scores, with the best ΔG being returned by (*S*)-**PB28** (− 145.7 kcal/mol), followed by (*S*)-**10** (− 143.61 kcal/mol), (*R*)-**16** (− 142.9 kcal/mol), and **14** (− 138.46 kcal/mol), in full agreement with their measured sub-nanomolar (**PB28**, **16**, **10**) and 1-digit nanomolar (**14**) S1R affinities. Additionally, IFD data suggests the presence of several interactions shared by all investigated ligands, including *(i)* an ionic interaction between a positively charged nitrogen atom and E172, a negatively charged residue, *(ii)* a cation-pi interaction involving the same nitrogen atom and F107, an aromatic residue, and (*iii*) several hydrophobic interactions with L182, L186, and, except for (*R*)-**16**, Y206. Although most of the interactions are shared among the simulated compounds, a distinct orientation can be observed when comparing the top-scored conformations of (*S*)-**PB28** and (*S*)-**10** with those returned by **14** and (*R*)-**16**. Specifically, (*S*)-**PB28** and (*S*)-**10** are predicted to position their methoxy group towards L95, thus forming a hydrophobic interaction. On the other hand, **14** and (*R*)-**16** direct their R-group (see Fig. 1) away from this residue. Interestingly, visual inspection suggests that a slight variation in terms of posing could potentially be responsible for a different conformation of the protein cavity. Indeed, an induced-fit rearrangement involving the α4 helix of the protein is observed upon binding with (*S*)-**PB28** and (*S*)-**10** only. Figure 2, showing the superposition of the **S1R-(S)-PB28** and **S1R-(R)-16** complexes returned by IFD simulations, provides a concrete idea of the observed slight α4 displacement. Notably, Schmidt et al.^22^ indicate that the conformation adopted by the α4 helix of S1R upon ligand binding is associated with its response (agonism vs. antagonism). More specifically, the authors report on the differences between the S1R crystal structure complexed with haloperidol (antagonist) and that complexed with pentazocine (agonist), highlighting that these two compounds induce two distinct α4 conformations, namely one closer to the binding site (haloperidol) and the other away from it (pentazocine). Importantly, **PB28** is herein predicted to induce a further α4 helix approach, consistently with its known S1R antagonist activity.

#### Molecular dynamics simulations

**Figure 3:**
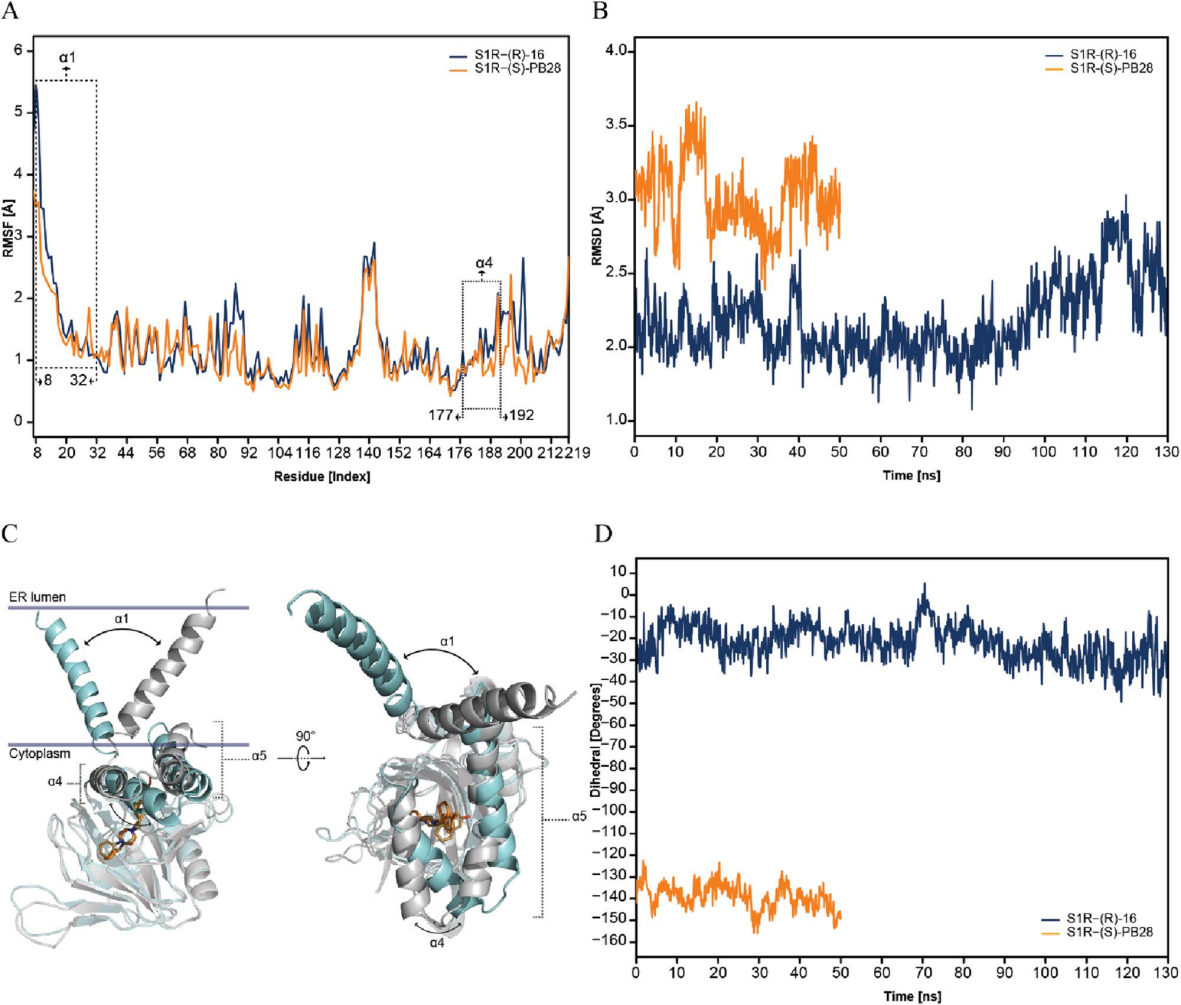
(**A**) RMSF of the alpha-carbon atoms belonging to each residue (Å) of the α4 helix of **S1R-(*R*)-16** (blue) and **S1R-(*S*)-PB28** (orange); (**B**) Time dependence of RMSD (Å) computed for the α4 helix of **S1R-(*R*)-16** (blue) and **S1R-(*S*)-PB28** (orange); (**C**) Superposition of selected snapshots of **S1R-(*R*)-16** **(gray)** and **S1R-(*S*)-PB28** (cyan) extracted from the performed MD simulations. The α1 and α4 helix shift is highlighted by a bidirectional black arrow; (**D**) Time dependence of the dihedral angle (d_α_) defined by the alpha-carbon atoms of the first (R8 and V177) and the last (T32 and S192) residues of α1 and α4. The initial equilibration trajectories were not included in the analysis.

Encouraged by these preliminary results and in an effort to support this hypothesis, two complexes returned by IFD simulations were used as the starting point for 200-ns long MD simulations, specifically, **S1R-(*S*)-PB28** and **S1R-(*R*)-16**. Notice that these complexes were selected since **(*S*)-PB28** and **(*R*)-16** represent ideal molecular probes. Indeed, despite their high S1R affinity and structural similarity (see Table 1) only one of them (i.e., (S)-**PB28**) acts as antiviral. As an initial step of the study, we computed the time-dependent Root Mean Square Deviation (RMSD) for all alpha-carbon atoms of the protein during the simulation. Figure S1 in the supporting information indicates that equilibration of the complexes required 70 ns (**S1R-(*R*)-16**) and 150 ns (**S1R-(*S*)-PB28**), and consequently, the last 130 ns and 50 ns were analyzed for **S1R-16** and **S1R-PB28**, respectively. Notably, average RMSD values of 0.97 Å ± 0.38 and 2.06 Å ± 0.38 were computed for **S1R-(*S*)-PB28** and **S1R-(*R*)-16**, respectively, indicating the reliability of the performed IFD simulations. Specifically, the interactions with E172 and F107 remained intact throughout the simulations of both **S1R-(*S*)-PB28** and **S1R-(*R*)-16**. Moreover, additional interactions involving the –OH substituent were predicted to be established with T202 and Y206, as shown in Figure S2 of the supporting information. Figure 3A reports the Root Mean Square Fluctuation (RMSF) values computed for the alpha-carbon atoms of each residue within the investigated systems, a higher level of flexibility can be observed in both α4 (residues 177–192) and α1 (residues 8–32) helixes of **S1R-(*R*)-16**, in comparison to **S1R-(*S*)-PB28**. Expanding on these findings, we computed the time-dependent RMSD values returned by the alpha-carbon atoms of the α4 helix (as shown in Fig. 3B). It is worth noting that our starting IFD complexes exhibited, between them, an initial shift of the α4 helix by 0.56 Å. The values obtained from our MD simulations indicate that this shift becomes more pronounced in time, and this protein portion can reach a different state depending on the bound ligand. This data again supports the robustness of the IFD docking data. Significantly, the observation that the α4 helix undergoes a conformational change, resulting in a different state induced by ligand-binding, is consistent with a recent study that reported results from MD simulations^51^.

In particular, Figure S3 (available in the supporting information) displays the superposition of the average conformations of **S1R-(*S*)-PB28** and **S1R-(*R*)-16** complexes as returned by the performed MD simulations. Remarkably, a shift of 1.8 Å involving the alpha-carbon atom belonging to A185 is observed after comparing the two complexes. This observation is in full agreement with the paper by Schmidt et al. (see Fig. 2C of reference 22) reporting the same conformational switch after comparing the x-ray crystal structures in complex with haloperidol and pentazocine. Furthermore, we computed the time-dependence of the dihedral angle defined by the alpha-carbon atoms of the first and the last residues of α1 and α4 (residues R8, T32, V177 and S192, hereinafter referred to as d_α_). As evident in Fig. 3C and 3D, during the simulation **S1R-(*S*)-PB28** reaches a very different conformational state (d_α_ ≈ − 140°) with respect to **S1R-(*R*)-16** (d_α_ ≈ − 30°), hence suggesting the presence of a conformational coupling involving these two helices. Noteworthy the visual inspection performed on the obtained trajectories indicates that such a difference is mostly due to a different orientation of the protein with respect to the membrane, rather than to a movement of α1 within the membrane. Such a significant conformation effect might represent a mechanical switch triggered by ligand binding. Whether this switch affects the oligomerization of S1R or its interactions with other membrane proteins or cytoplasmic biomolecules remains to be ascertained. Notably, our data support the physiological relevance of α1 whose conformation was recently hypothesized to be crucial for the closed-to-open transition of the protein^51^.

## Conclusions

As recently reviewed, S1R may play a critical role in early-stage virus replication^17^. In addition to the evidence already described, further support for S1R as a potential target for antiviral action is provided by the following observations: *i)* S1R is enriched in lipid rafts where it colocalizes with viral replicative proteins, such as NSP6; *ii)* viruses use stress response mechanisms to promote their replication, and S1R regulates stress response^20,52,53^; *iii)* Activation of S1R by its ligands induces autophagy, a mechanism used by some viruses including coronaviruses to evade the host immune system^52,54^; *iv)* several drugs intended for different uses were screened against viruses (e.g.; EBV, HCV, Ebola, Influenza H5N1), and drugs associated with antiviral activity were also S1R^17^*.* Based on our findings and the evidence that pan-viral disease mechanisms were revealed through the comparison of host-coronavirus protein interaction networks, we have tested a chemical library of S1R ligands. Some returned anti-SARS-CoV-2 effects, but we have not found a clear correlation between their S1R affinity and antiviral activity. The phospholipidosis effect has been proposed as an explanation for the lack of in vivo activity of some S1R ligands in preclinical studies, and we cannot rule it out. However, the correlation of the chemical properties of the compounds tested with the antiviral activity suggests that the phospholipidosis effect is not responsible for the observed effects. Very similar compounds in terms of structure, pKa and logP (see Table S1 in the Supporting Information) would exert the same effect, which is not the case. Compounds **13** and **14** give one representative example with identical physico-chemical properties and opposite anti-SARS-CoV-2 activity. Additionally, the phospholipidosis hypothesis contrasts with the genomic data and retrospective/prospective clinical studies mentioned above. Even though the effect of S1R ligands on phospholipidosis remains to be tested, our investigation has focused on the conformational changes these ligands may induce in the S1R protein, which may affect its interactions with client proteins. Recent bioluminescence resonance energy transfer (BRET) assays have shown that different S1R responses occur based on receptor multimerization, depending on the binding of structurally diverse ligands^38^. Our preliminary data support the hypothesis that shedding light on the conformational effects induced by ligand binding is crucial for the rational design of S1R ligands. Therefore, despite the complexity of the framework, our findings suggest that modulation of S1R could still be a viable approach to combat SARS-CoV-2 and SARS-CoV-1 infections as well as other highly pathogenic viruses.

### Supplementary Information

Supplementary Information.

## Data Availability

All data are reported in the manuscript and the SI provided.

## Abbreviations

(S1R): Sigma-1 receptor
(CNS): Central nervous system
(SARS): Severe acute respiratory syndrome
(MERS): Middle East respiratory syndrome
(COVID-19): Coronavirus disease 2019
(FDA): Food and Drug Administration
(S2R): Sigma-2 receptor
(KO): Knocking out
(KD): Knocking down
(IFD): Induced Fit Docking
(MD): Molecular Dynamics
(RMSD): Root Mean Square Deviation
