# Supplementary material for: A conformational rearrangement of the SARS-CoV-2 host protein sigma-1 is required for antiviral activity: insights from a combined in-silico/in-vitro approach
Source: Sci Rep. 2023 Aug 7;13:12798. doi: 10.1038/s41598-023-39662-w (PMC10406941; doi:10.1038/s41598-023-39662-w)
Supplement: Supplementary file 1 — Supplementary Information. [file 41598_2023_39662_MOESM1_ESM.docx]

**A conformational rearrangement of the SARS-CoV-2 host protein sigma-1 is required for antiviral activity: insights from a combined in-silico/in-vitro approach**

Francesca Serena Abatematteo,*^1^* Pietro Delre,*^2^* Ivan Mercurio,*^2,3^* Veronica Rezelj,^4^ Dritan Siliqi,^2^ Stephanie Beaucourt,^4^ Gianluca Lattanzi,^5,6^ Nicola Antonio Colabufo,*^1^* Marcello Leopoldo,*^1^* Michele Saviano,^7^ Marco Vignuzzi,^4,8^ Giuseppe Felice Mangiatordi,*^2^*^*^ Carmen Abate*^1,2^**

*^1^Dipartimento di Farmacia-Scienze del Farmaco, Università degli Studi di Bari Aldo Moro Via Orabona, 4 79125 BARI, Italy;*

*^2^Consiglio Nazionale delle Ricerche (CNR), Istituto di Cristallografia, Via Amendola 122/O, 70126, Bari, Italy*

*^3^Department of Environmental, Biological and Pharmaceutical Sciences and Technologies, University of Campania “Luigi Vanvitelli”, Via Antonio Vivaldi 43, 81100 Caserta, Italy*

*^4^Institut Pasteur, Viral Populations and Pathogenesis Unit, CNRS UMR 3569, Paris, France*

*^5^Department of Physics, University of Trento, Via Sommarive 9, 38123, Povo-Trento, Italy*

*^6^TIFPA Trento Institute for Fundamental Physics and Applications, Via Sommarive 9, 38123, Povo-Trento, Italy*

*^7^Consiglio Nazionale delle Ricerche (CNR), Istituto di Cristallografia, Via Vivaldi 43, 81100, Caserta, Italy*

^8^*A*STAR Infectious Diseases Labs (A*STAR ID Labs), Agency for Science, Technology and Research (A*STAR), 8A Biomedical Grove, Immunos #05-13, Singapore 138648, Singapore*

**Table of content**

**Figure S1.** Time-dependence of RMSD (Å) computed for the investigated **S1R-(*S*)-PB28** (blue line) and **S1R-(*R*)-16** (orange line) complexes.

**Figure S2**. Selected snapshots showing the binding mode of A) (*S*)-**PB28** and B) (*R*)-**16** within the binding site of S1R. For the sake of clarity, only polar hydrogen atoms are shown. Important residues are rendered as sticks while the proteins are represented as cartoon. Salt-bridge, cation-pi, and H-bond interactions are depicted by red, green and black dotted lines, respectively.

**Figure S3**. Superposition of the average conformations **of S1R(cyan)-(S)-PB28(orange)** and **S1R(gray)-(R)-16 (sand)** complexes as returned by the performed MD simulations. The shift of the alpha carbon atom belonging to A185 is depicted by a bidirectional black arrow. For the sake of clarity, only polar hydrogen atoms are shown. Important residues are rendered as sticks while the proteins are represented as cartoons.

**Table 1.** pKa values and octanol/water partition coefficients (logP) computed by Qikprop for the compounds under investigation.

**Figure S1.**


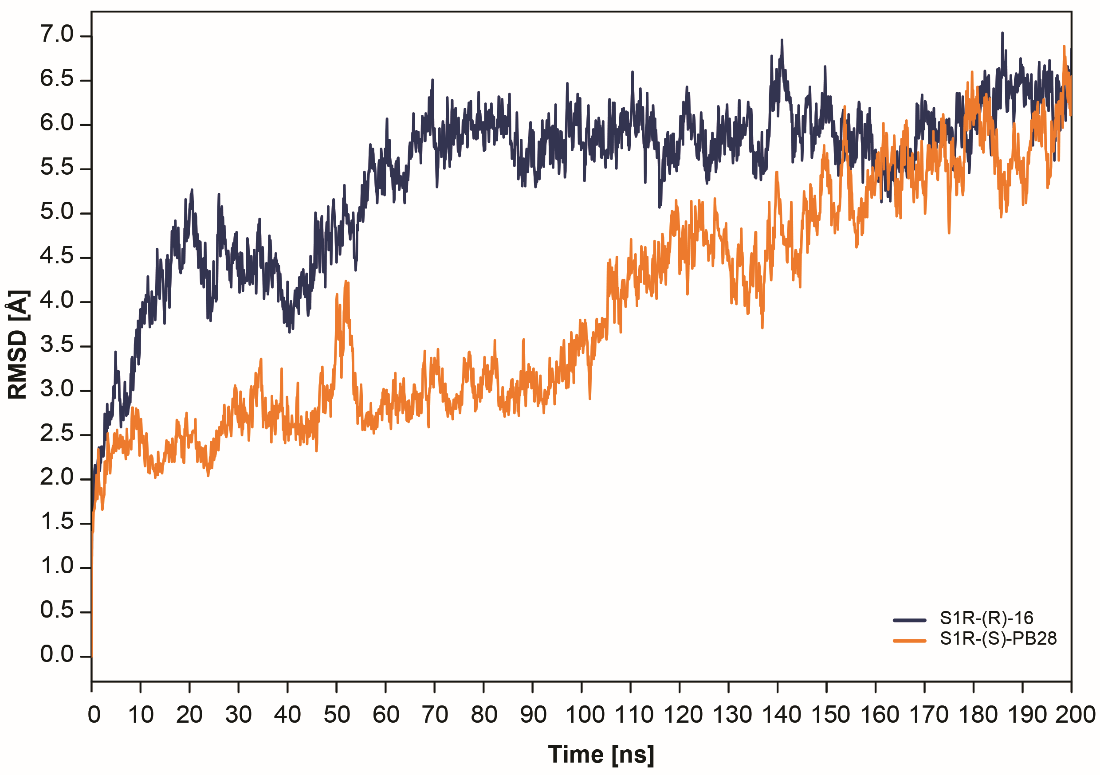


**Figure S2.**


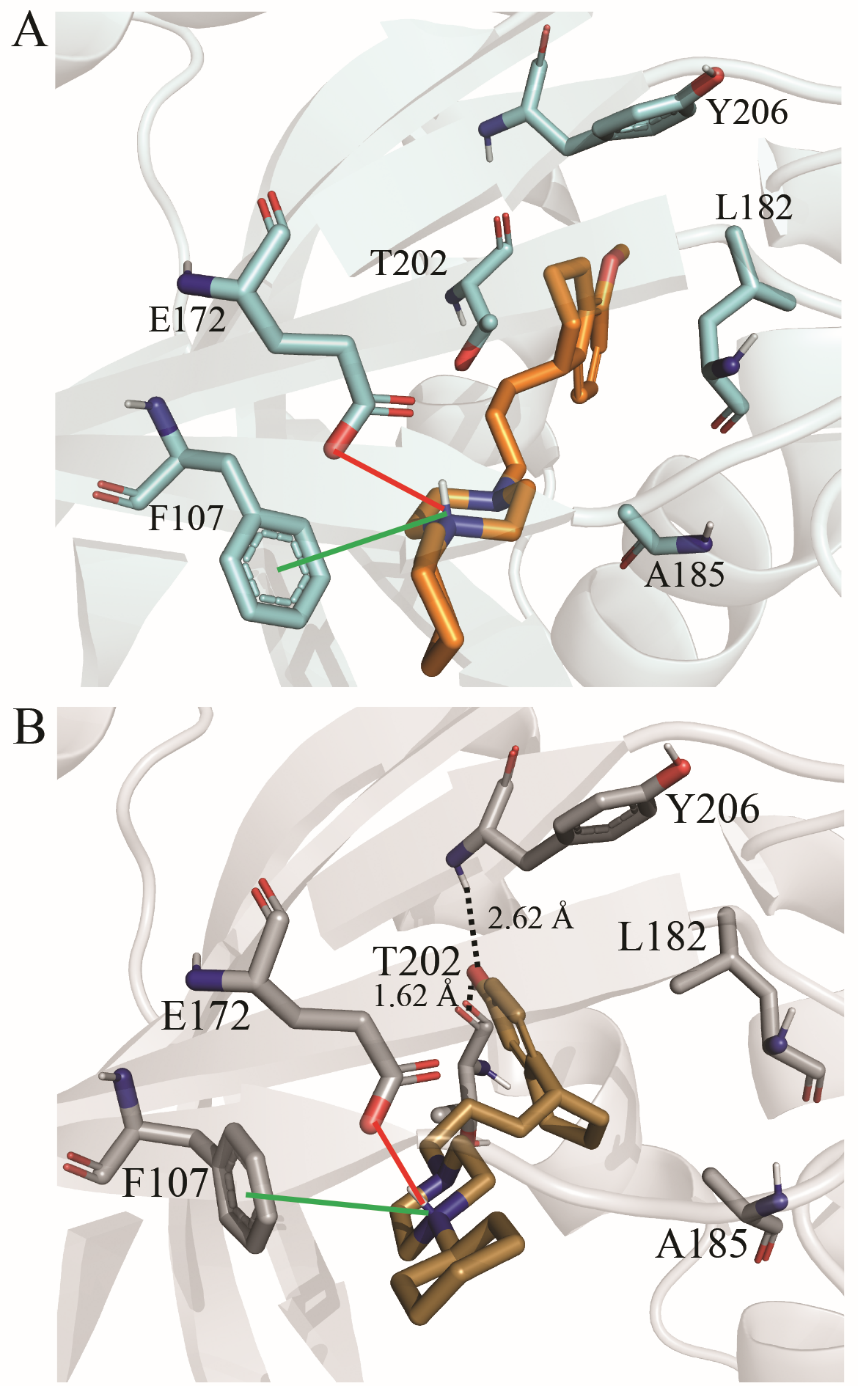


**Figure S3.**

**
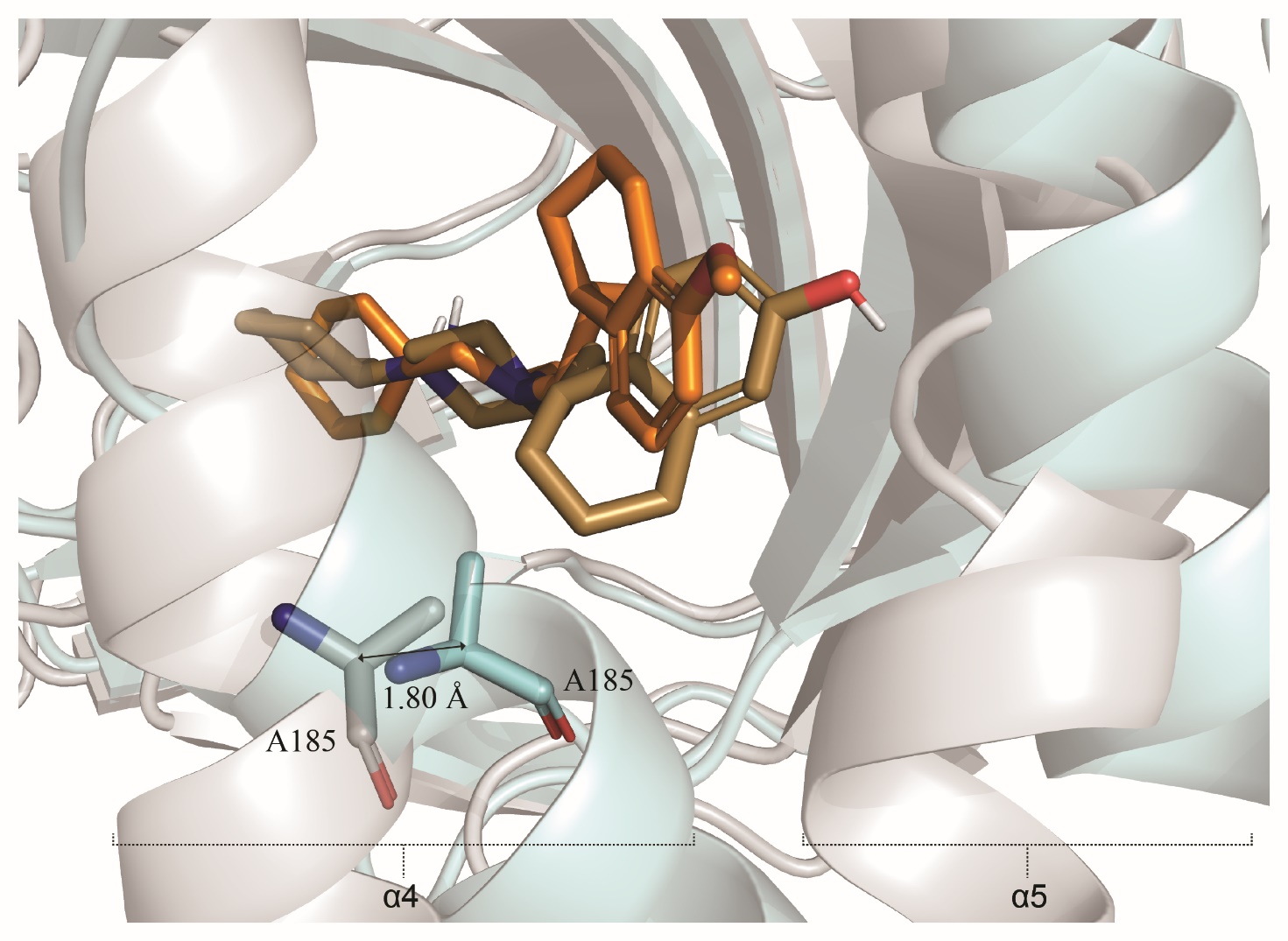
**

**Table 1.**

| **Compound** | **QPlogPo/w** | **macro-pKa** |
| --- | --- | --- |
| **PB28** | 4.82 | 5.32-9.15 |
| **1** | 3.64 | 4.08-9.10 |
| **2** | 4.04 | 4.03-9.32 |
| **3** | 4.37 | 4.59-9.25 |
| **4** | 5.21 | 5.53-9.15 |
| **5** | 5.51 | 5.55-9.15 |
| **6** | 5.84 | 5.55-9.15 |
| **7** | 4.69 | 5.32-9.15 |
| **8** | 4.98 | 5.53-9.15 |
| **9** | 5.49 | 5.55-9.15 |
| **10** | 4.70 | 5.32-9.15 |
| **11** | 4.56 | 4.52-9.24 |
| **12** | 5.29 | 5.54-9.15 |
| **13** | 4.72 | 4.52-9.24 |
| **14** | 4.61 | 4.52-9.24 |
| **15** | 4.13 | 5.32-9.13-10.50 |
| **16** | 3.99 | 5.32-9.12-10.42 |
| **17** | 3.84 | 4.52-8.93-9.53 |
| **18** | 3.90 | 4.52-8.92-9.52 |
| **19** | 3.99 | 7.69 |
| **20** | 4.15 | 7.69 |
| **21** | 3.91 | 7.69 |
| **23** | 3.30 | 4.53-8.68 |
| **24** | 4.04 | 5.15-8.83 |
| **25** | 4.72 | 5.33-9.24 |
| **26** | 3.48 | 8.23-9.17-13.31 |
| **27** | 3.39 | 7.59-9.21 |
| **28** | 3.86 | 4.58-9.08 |
| **29** | 4.14 | 5.32-9.15 |
| **30** | 4.08 | 4.03-9.04-12.92 |
| **31** | 4.26 | 4.04-9.20 |
| **32** | 5.10 | 4.16-9.28 |
| **33** | 3.69 | 5.51-9.16 |
| **34** | 2.78 | 4.39-9.26 |
| **35** | 1.50 | 4.58-9.12 |
| **36** | 5.24 | 5.30-8.77 |
| **37** | 5.26 | 5.30-8.77 |
| **38** | 5.39 | 5.30-8.77 |
| **F190** | 5.16 | 10.60 |
| **PB212** | 4.96 | 10.50 |
| **39** | 4.73 | 9.16 |
